# ﻿*Mukariasakaeratensis* sp. nov. (Hemiptera, Cicadellidae, Deltocephalinae), a new species of bamboo leafhopper from Sakaerat Biosphere Reserve, Thailand

**DOI:** 10.3897/zookeys.1239.145803

**Published:** 2025-05-28

**Authors:** Kanyakorn Piraonapicha, Nithina Kaewtongkum, Narin Chomphuphuang, Panrak Kimsawat, Kittisak Kumtanom, Yudthana Samung

**Affiliations:** 1 Queen Sirikit Botanic Garden, The Botanical Garden Organization, Chiang Mai 50180, Thailand Queen Sirikit Botanic Garden, The Botanical Garden Organization Chiang Mai Thailand; 2 Sakaerat Environmental Research Station, Thailand Institute of Scientific and Technological Research, Wang Nam Khieo District, Nakhon Ratchasima 30370, Thailand Thailand Institute of Scientific and Technological Research Nakhon Ratchasima Thailand; 3 Department of Entomology and Plant Pathology, Faculty of Agriculture, Khon Kaen University, Khon Kaen 40002, Thailand Khon Kaen University Khon Kaen Thailand; 4 Department of Integrated Engineering (Intelligent Agricultural Engineering), Faculty of Engineering, Pathumwan Institute of Technology, Bangkok, 10330, Thailand Pathumwan Institute of Technology Bangkok Thailand; 5 Department of Medical Entomology, Faculty of Tropical Medicine, Mahidol University, Bangkok 10400, Thailand Mahidol University Bangkok Thailand

**Keywords:** Barcoding gene, COI, deciduous dipterocarp forests, identification key, molecular identification, morphology, Mukariini, Thailand, *Vietnamosasa*

## Abstract

*Mukariasakaeratensis* Piraonapicha & Chomphuphuang, **sp. nov.** is described based on male and female specimens recently collected in Nakhon Ratchasima, Thailand. The new species is herein described by an integrative approach combining morphological and molecular evidence. Genetic distance analyses revealed a potential barcoding gap (K2P) of 0.20–12.07% for COI in *Mukaria*. Species delimitation methods ABGD and ASAP demonstrated promising results for the COI gene. This species clearly differs from all its congeners in the aedeagal shaft abruptly narrowed and curved inward in the distal half, and with a pair of spines pointed anteriorly. *Mukariasakaeratensis***sp. nov.** has been found on the bamboo *Vietnamosasapusilla* (A. Chev. & A. Camus) T.Q. Nguyen. This finding constitutes the first recorded instance of a specialized member of the tribe Mukariini (Hemiptera: Cicadellidae: Deltocephalinae) feeding exclusively on bamboo from the genus *Vietnamosasa*. The holotype has been deposited in the Entomology Section, Queen Sirikit Botanic Garden, The Botanical Garden Organization, Thailand.

## ﻿Introduction

The leafhopper genus *Mukaria* was established by Distant in 1908 based on the type species *Mukariapenthimioides* from Sri Lanka. Currently, the genus consists of 15 valid described species reported in Bangladesh, China, India, Indonesia, Japan, Sri Lanka, Thailand, and Pakistan (Distant 1908; Matsumura 1912; Ishihara 1961; Hayashi 1996; Cai and Ge 1996; Yang and Chen 2011; Viraktamath and Webb 2019; Yao et al. 2019; Zhao et al. 2024). The bamboo-feeding leafhoppers belonging to the genus *Mukaria* are highly specialized herbivores with distinct roles in their ecosystems, both as herbivores and disease vectors. Their remarkable adaptations, including unique morphological traits and color polymorphism, highlight their evolutionary responses to the bamboo-dominated environments they inhabit (Ramaiah et al. 2023; Zhao et al. 2024).

In this study, we describe a new species, *Mukariasakaeratensis* sp. nov., discovered at the Sakaerat Environmental Research Station within the Sakaerat Biosphere Reserve, Nakhon Ratchasima Province, in northeastern Thailand. This species is characterized by its dark-colored body and was found exclusively on its host plant, the small-sized sympodial bamboo *Vietnamosasapusilla* (A.Chev. & A.Camus) T.Q.Nguyen, in dry dipterocarp forests. The description of *Mukariasakaeratensis* sp. nov. is based on an integrative approach, combining detailed morphological analyses of both male and female specimens with molecular evidence derived from COI barcoding, which provides robust DNA-based support for its classification.

## ﻿Material and methods

### ﻿Specimen collection and morphological study

The *Mukaria* specimens were collected from a restoration forest within deciduous dipterocarp forests, Sakaerat Biosphere Reserve, Nakhon Ratchasima Province, Thailand. The new species was found on leaves of *Vietnamosasapusilla* (A.Chev. & A.Camus). Specimens of the new species were prepared through dry pinning and preservation in alcohol for morphological examination. The genitalia of males and females were dissected from the abdominal segment. The dissected genitalia were cleared in a 10% potassium hydroxide (KOH) solution for one day at room temperature, washed with distilled water, and then stored in glycerine in microvials before examination and imaging. Morphological observations were conducted using a Nikon SMZ445 stereomicroscope. Images were taken using a Nikon Digital Sight Ri1 camera attached to a Nikon AZ100M stereomicroscope and processed with NIS-Elements-D for a multi-focused montage. Images of genitalia and wing venation were taken using a Nikon DS-F12 camera attached to a Nikon Eclipse Ci-L compound microscope. Images of living workers and nest entrances were taken using a Canon RF100 mm f/2.8L macro lens attached to a Canon R6 digital camera. Specimens are deposited in the Entomology Section, Queen Sirikit Botanic Garden, The Botanical Garden Organization, Chiang Mai Province, Thailand (**QSBG**); Department of Entomology and Plant Pathology, Faculty of Agriculture, Khon Kaen University, Khon Kaen Province, Thailand (**KKU**); Sakaerat Environmental Research Station, Thailand Institute of Scientific and Technological Research, Wang Nam Khieo District, Nakhon Ratchasima Province, Thailand (**SERS**); and the Thailand Natural History Museum of the National Science Museum, Pathum Thani **(THNHM)**.

### ﻿DNA extraction and PCR amplification

Tissue samples from the right legs of male and female adults were preserved in 95% ethanol. Total DNA was extracted following the DNeasy Blood & Tissue Kit (Qiagen) protocol, and the extracted DNA was stored at -20 °C. The PCR reaction mix had a total volume of 50 μl, comprising 20 μl ultrapure water, 3 μl of the DNA template, 1 μl of each primer (10 μM), and 25 μl of master mix. Thermal cycling started with incubation at 94 °C for 1 min, followed by 40 cycles at 94 °C for 30 s, annealing at 48, 50 °C for 50 s, and extension at 72 °C for 1 min, with a final extension step at 72 °C for 5 min. All PCR products were visualized on 1.5% agarose gels using Omnipur Agarose (United States of America). A 592 bp fragment of the COI gene was amplified using universal primers LCO1490 and HCO2198, as described by Folmer et al. (1994).

### ﻿DNA sequencing and analyses

Purified PCR products underwent bidirectional Sanger sequencing on the ABI PRISM 3130x.1 Genetic Analyzer (Applied Biosystems, Foster City, USA) Sequencing was performed by Macrogen Inc. Sequencing (Seoul, Korea). Consensus sequences were generated, and sequences of the candidate DNA barcodes aligned using ClustalW and verified using BIOEDIT v. 7.2.5 (Hall 1999). Pairwise distances between COI sequences were calculated using MEGA v. 10 (Tamura et al. 2021) using the “Distances” option and “Nucleotide: *p*-distance” model option for distances. The COI sequences are available on GenBank (https://www.ncbi.nlm.nih.gov/genbank) and the Barcode of Life Data System (BOLD) (https://v3.boldsystems.org/) under accession numbers specified in Table 1.

**Table 1.** Species and specimen information used in the DNA barcoding analysis.

Species	Locality	Sex	Voucher	Accession number/ sequence ID	Sources
*M.sakaeratensis* Piraonapicha & Chomphuphuang, sp. nov.	Nakhon Ratchasima, Thailand	Female	QSBG-2024-0046-0018	PP582202	This study
	Nakhon Ratchasima, Thailand	Female	QSBG-2024-0046-0019	PP582203	This study
	Nakhon Ratchasima, Thailand	Female	QSBG-2024-0046-0020	PP582204	This study
	Nakhon Ratchasima, Thailand	Female	QSBG-2024-0046-0021	PP582205	This study
	Nakhon Ratchasima, Thailand	Male	QSBG-2024-0046-0008	PP582206	This study
	Nakhon Ratchasima, Thailand	Male	QSBG-2024-0046-0009	PP582207	This study
	Nakhon Ratchasima, Thailand	Male	QSBG-2024-0046-0010	PP582208	This study
	Nakhon Ratchasima, Thailand	Male	QSBG-2024-0046-0011	PP582209	This study
*M.splendida*	Yunnan, China	Male		MG813485	Yang and Dai 2021
				MK862276	GenBank
				MW487892	GenBank
	India	Male		OM869458	Ramaiah et al. 2023
	India	Male		OM345004	Ramaiah et al. 2023
	India	Male		OP617462	Ramaiah et al. 2023
	India	Male		OP616038	Ramaiah et al. 2023
	Punjab, Pakistan			GMPJA9077-21.COI-5P	BOLD Systems
	Punjab, Pakistan			GMPJA609-21.COI-5P	BOLD Systems
	Punjab, Pakistan			GMPJA5825-21.COI-5P	BOLD Systems
	Punjab, Pakistan			GMPJA4951-21.COI-5P	BOLD Systems
	KwaZulu-Natal, South Africa			SAKZA3134-22.COI-5P	BOLD Systems
	Chittagong, Bangladesh			GMBDE4119-23.COI-5P	BOLD Systems
*M.maculata*				MG736687	GenBank
				MG736688	GenBank
				MG736689	GenBank
				MG736690	GenBank
*M.albinotata*				MG736685	GenBank
				MG736686	GenBank
*M.bambusana*				MG736694	GenBank
				MG736695	GenBank
				MG736696	GenBank
				MG736697	GenBank
*Deltocephalusvulgaris*	Hainan, China			MT998308	Wu et al. 2022

To assess phylogenetic relationships, COI sequences from previous studies were retrieved from GenBank, as detailed in Table 1. Sequence alignment for each individual gene was performed using the ClustalW algorithm (Thompson et al. 1994) with default settings in MEGA 11 software (Tamura et al. 2021). To determine the most suitable nucleotide substitution models, jModelTest v. 2.1 (Darriba et al. 2012) was employed, utilizing the likelihood algorithm and evaluating 11 schemes encompassing 88 potential models. The optimal model was chosen based on the Akaike Information Criterion (AIC), resulting in the selection of GTR+I. For the phylogenetic reconstructions, *Deltocephalusvulgaris* (MT998308) was used as the outgroup to root the trees.

Two methods were used for phylogenetic analysis: maximum likelihood (ML) and Bayesian inference (BI). The ML reconstructions were performed using RAxML-NG (Kozlov et al. 2019) on the CIPRES Science Gateway (v. 1.2.0) (Miller et al. 2010), with 1000 bootstrap replicates to assess support. For the BI analysis, MrBayes v. 3.2.7 (Ronquist et al. 2012) was utilized, applying the same optimal substitution model identified in the ML analysis. The BI analysis involved running four parallel Markov Chain Monte Carlo (MCMC) chains for 10 million generations, with trees saved every 500 generations. To ensure convergence, we conducted several diagnostic tests in addition to discarding the initial 25% of the posterior distribution trees as burn-in. Convergence was assessed by examining the consistency of posterior probabilities across chains and calculating effective sample sizes (ESS) for key parameters using Tracer v. 1.7. ESS values exceeded 200 for all parameters, indicating adequate sampling and mixing of chains. Furthermore, the average standard deviation of split frequencies was monitored and found to be below 0.01, confirming convergence as per MrBayes recommendations. Visual inspection of trace plots for likelihood values and posterior probabilities confirmed stationarity and consistent mixing across chains. The final consensus tree was constructed using the 50% majority rule from the remaining trees. The Automatic Barcode Gap Discovery (ABGD) method (Puillandre et al. 2012) was utilized through an online web-based interface accessible at https://bioinfo.mnhn.fr/abi/public/abgd/. The analysis parameters were configured to default settings, employing tree comparison models including Jukes-Cantor (JC69), Kimura (K2P), and Simple Distance. Additionally, Assemble Species by Automatic Partitioning (ASAP) was run on the server https://bioinfo.mnhn.fr/abi/public/asap/asapweb.html, utilizing a substitution model to compute distances similar to ABGD.

## ﻿Results

### ﻿Taxonomy

#### ﻿Genus Mukaria

Taxon classificationAnimaliaHemipteraCicadellidae

Distant, 1908

07A3C215-2469-5971-90C0-3ED41A9E5695

Mukaria Distant, 1908: 269. Type species: Mukariapenthimioides Distant, 1908, by original designation.
Parabolotettix Matsumura, 1912: 280.Type-species: Parabolotettixmaculatus Matsumura, 1912, by original designation. Synonymised by Schumacher 1915: 97.
Ikomella Ishihara, 1961: 253. Type species: Ikomellaconfersa Ishihara, 1961, by original designation. Synonymised by Linnavuori 1979: 985.

##### Description.

Revised from Viraktamath and Webb (2019). ***Adult.*** In full-face view, head broader than long (including eye margins), with upper portion appearing swollen in lateral view; frontoclypeus flat, horizontal, gradually expanding dorsally; clypellus parallel sides, convex, and extends beyond typical curvature of gena; lorum elongated and narrow; antennae originate near the upper corners of the eyes in frontal view, with pedicel visible from dorsal view. Tentorium with anterior arm thin, dorsal arm about as thin and half as long as anterior arm, arising at mid-length. Pronotum convex, with faint transverse rugosity, twice as wide as long medially, about as long as mesonotum, lateral margins carinate, convex anterior margin, slightly concave posterior margin. Mesonotum with faint transverse rugosity and granulose basal triangles. Forewing with separate claval veins, 3 subapical cells, open inner subapical cell, non-confluent veins R_4+5_ and M_1+2_. Hind wing with a marginal vein, either complete or discontinuous near apical region. ***Male genitalia.*** Male pygofer depressed, with setae in distal lower half; posterior margin of tergum IX shallowly U-shaped to receive abdominal segment X, with ventral marginal process variously developed (absent in *M.omani*). Subgenital plate with scattered setae on ventral surface and without apical membranous appendage. Style with well-developed lateral lobe, apophysis short, narrowed distally, surface sculptured. Connective and aedeagus fused, arms widely placed, parallel to each other. Aedeagus with two shafts fused in basal 0.2 to 0.5, then divergent, each with processes near apical gonopore. ***Female genitalia.*** Sternite VII approximately as long as VI, concave posterior margin. Valvula I more or less straight, strigate to concatenate (with sculpturing elements or scales fused to one another), or imbricate (with overlapping scales) ventrally at 1^st^ valvula apex, occupying slightly less than 0.5 distally, strigae oblique. In lateral view, valvula II almost straight, toothed area not preceded by either hyaline area or prominent tooth, occupying distal 0.5; tooth prominent, well separated from each other, without secondary dentition.

##### Species and distribution of *Mukaria*.

*M.albinotata* Cai & Ge, 1996: China (Cai and Ge 1996; Yang and Chen 2011)

*M.creagra* Zhao, Luo & Chen, 2024: China (Zhao et al. 2024)

*M.confersa* (Ishihara, 1961): Thailand (Ishihara 1961, Yang and Chen 2011)

*M.flavida* Cai & Ge, 1996: China (Cai and Ge 1996; Yang and Chen 2011)

*M.hainanensis* Yao, Yang & Chen, 2019: China (Yao et al. 2019)

*M.lii* Yang & Chen, 2011: China (Yang and Chen 2011)

*M.maculata* (Matsumura, 1912): China, Japan and Indonesia (Matsumura 1912; Hayashi 1996; Yang and Chen 2011);

*M.nigra* Kuoh & Kuoh, 1983: China (Yang and Chen 2011)

*M.omani* Viraktamath & Webb, 2019: India (Viraktamath and Webb 2019)

*M.penthimioides* Distant, 1908: Sri Lanka and India (Distant 1908; Yang and Chen 2011, Viraktamath and Webb 2019)

*M.splendida* Distant, 1908: Bangladesh, India and Pakistan (Distant 1908; Yang and Chen 2011, Viraktamath and Webb 2019, Ramaiah et al. 2023)

*M.striola* Zhao, Luo & Chen, 2024: China (Zhao et al. 2024)

*M.vakra* Viraktamath & Webb, 2019: India (Viraktamath and Webb 2019)

*M.variabilis* Evans, 1973: Indonesia (New Guinea) (Evans 1973; Yang and Chen 2011)

*M.zonata* Hayashi, 1996: Japan (Hayashi 1996; Yang and Chen 2011)

#### ﻿ Mukaria sakaeratensis

Taxon classificationAnimaliaHemipteraCicadellidae

Piraonapicha & Chomphuphuang sp. nov.

2C7027DD-0C8B-5467-9B52-AD5D66F6BF5C

https://zoobank.org/0148A5E2-30B3-48DE-A13C-F2EB093EDDA3

Figs 3 , 4 , 5 , 6

##### Type material examined.

**Thailand. *Holotype*** • One male, Sakaerat Biosphere Reserve, Udomsub Sub-District, Wang Nam Khiao District, Nakhon Ratchasima Province, Thailand, 14°30'33.7"N, 101°56'23.4"E, 326 m a.s.l., 10.VI.2024, N. Kaewtongkum leg. (QSBG-2024-0046-0001); ***Paratypes*** • 10 males, same date, locality, and collector as holotype (QSBG-2024-0046-0002 to QSBG-2024-0046-0011) • 10 female, same date, locality and collector as holotype (QSBG-2024-0046-0012 to QSBG-2024-0046-0021) • 10 males, same date, locality, and collector as holotype (KKU-AG-I-0001 to KKU-AG-I-0010) • 10 female, same date, locality and collector as holotype (KKU-AG-I-0011 to KKU-AG-I-0020) • 5 males, same date, locality, and collector as holotype (SERS -I-H-2024-0001 to SBR-I-H-2024-0005) • 5 female, same date, locality and collector as holotype (SERS -I-H-2024-0001 to SBR-I-H-2024-0010) • 5 males, same date, locality, and collector as holotype (THNHM-I-00030082 to THNHM-I-00030086) • 5 females, same date, locality, and collector as holotype (THNHM-I-00030087 to THNHM-I-00030091).

##### Description.

***Measurements.* Male.** Body length (including tegmen) 2.92 ± 0.12 mm (*N* = 10); head widths 0.87 ± 0.02 mm (*N* = 10). **Female.** Body length (including tegmen) 3.07 ± 0.11 mm (*N* = 10); head widths 0.92 ± 0.03 mm (*N* = 7).

***Coloration.* Male.** Head (excluding eyes) entirely black in dorsal view, postclypeus dark brown with median area brown; lorum and gena dark brown; pronotum and scutellum dark brown; forewing dark brown, apical 1/4 with brown, yellow spot at mid-length extending to lateral margin of pronotum, oblique spot on costa, subtriangular spot near outer apical cell, small yellow spot near ScP+RA, hindwing brown, hyaline; wing veins brown; coxa and trochanter of all legs dark brown, femur, tibia and tarsi of fore and middle leg yellow; femur and tibia of hindleg brown, apex of tibia dark brown; basal half of 1 segmented tarsi segment pale brown, apical half dark brown, 2 segmented of tarsi pale brown, 3 segmented of tarsi and claw dark brown. **Female.** Similar to male.

***Male genitalia.*** In lateral view, male pygofer subtriangular, approximately twice as length as high. In ventral view, valve subtriangular and wider than long and with anterior margin slightly concave and posterior margin produced medially. In ventral view, subgenital plate subrounded, with subtriangular shaped apex, inner margin roundly convex, outer margin almost straight, slightly concave near apex, basal part of subgenital plate with approximately 12 long setae, and distal part with 9–13 long setae. Style, slightly wider at base with lateral process, preapical lobe rectangular, strongly concave near apex. Apex of style digitiform and curved outward. Connective Y shaped, stem as long as arms. Aedeagus with a pair of arcuate laterobasal processes, nearly 1/3 as long as aedeagal shaft, and aedeagal shaft abruptly narrowed and curved inward in distal half, half portion in ventral view with a pair of spines pointed anteriorly. In lateral view, near apex of aedeagus with strong subtriangular lobe pointed anteriorly (Fig. 5).

**Figure 1.**
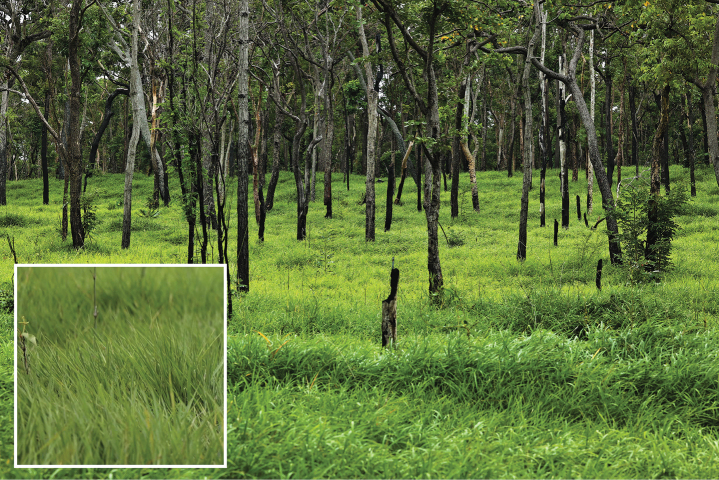
Deciduous dipterocarp forests, Sakaerat Environmental Research Station, Nakhon Ratchasima Province, Thailand, where the specimens of *Mukariasakaeratensis* Piraonapicha & Chomphuphuang, sp. nov. were collected.

***Female genitalia.*** Female sternite VII subrectangular, approximately 2 times longer than wide, with pair subtriangular lobes at apex, outer margin convex and strongly emarginate in middle. Valvula I almost straight. Valvula II similar to valvula I, but 1/3-part upper margin serrate. Pygofer spinose in posterior half, ovipositor not exceeding pygofer (Fig. 6).

##### Distribution.

**Figure 2.**
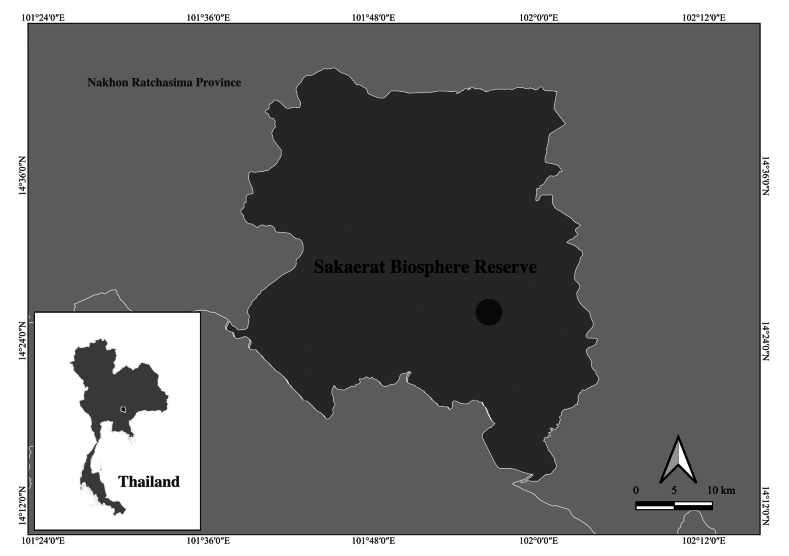
Map showing Sakaerat Biosphere Reserve, Nakhon Ratchasima Province, Thailand; black spot = sampling locality.

Thailand (Nakhon Ratchasima Province) (Fig. 2).

##### Etymology.

The specific epithet ‘*sakaeratensis*’ refers to the type locality.

##### Habitat.

The bamboo species *Vietnamosasapusilla* (A.Chev. & A.Camus) T.Q. Nguyen has been identified as the host plant for *Mukariasakaeratensis* sp. nov., a newly described species of bamboo-feeding leafhopper discovered in the Sakaerat Biosphere Reserve, Thailand. This finding marks the first documented ecological relationship between the bamboo genus *Vietnamosasa* and members of the tribe Mukariini (Hemiptera: Cicadellidae: Deltocephalinae), which are specialized herbivores feeding exclusively on bamboos. The genus *Vietnamosasa* comprises sympodial bamboos found in Southeast Asia, including Thailand, Cambodia, Laos, and Vietnam. *Vietnamosasapusilla*, commonly found in dry dipterocarp forests, is characterized by its small size and adaptation to fire-prone environments. During the collection of the new species of *Mukaria*, the plant height, measured from the base of the stem (at the soil surface) to the tallest part of the plant, was approximately 70–90 cm.

##### DNA barcode data.

In this study, genetic distances were calculated for the cytochrome *c* oxidase subunit I (COI) gene across several *Mukaria* species. The intraspecific genetic distances ranged from 0.10% to 0.20%, indicating relatively low genetic variation within individual species. When comparing between species, the interspecific genetic distances varied considerably, with the highest value of 23.71% observed between *M.bambusana* and *M.maculata* (2). Conversely, the lowest interspecific genetic distance was 12.07%, found between *M.sakaeratensis* sp. nov. and *M.splendida* (Fig. 7). These analyses revealed a clear barcoding gap (K2P) of 0.20–12.07% for the COI gene in *Mukaria*, demonstrating that this gene region effectively discriminates between different species within this genus while maintaining consistency within species. This substantial genetic differentiation supports the taxonomic distinctiveness of these *Mukaria* species, particularly the newly described *M.sakaeratensis*. In the maximum likelihood and Bayesian inference analyses, as well as the ABGD and ASAP methods, the new species was clearly distinct from other species included in the analysis (Fig. 8). Based on the COI phylogenetic tree, the new species was found in Indochina, while its closely related species, *M.maculata*, has been reported in Japan, Indochina, and Sundaland (Matsumura 1912; Hayashi 1996; Yang and Chen 2011). The phylogenetic analysis revealed strong branch support for the distinctiveness of the new species, highlighting its divergence from other species within the genus. Geographic distribution patterns indicate that most species of *Mukaria* have limited ranges, except for *M.maculata*, which exhibits a broader distribution across multiple biogeographic regions. This wide range suggests potential cryptic diversity within *M.maculata*, warranting further examination to determine whether populations from different regions represent distinct species. Additionally, the barcoding gap (K2P) observed in genetic distance analyses underscores the utility of COI for distinguishing species within *Mukaria*. These findings provide valuable insights into the phylogenetic relationships and biogeographic structure of this genus.

**Figure 3.**
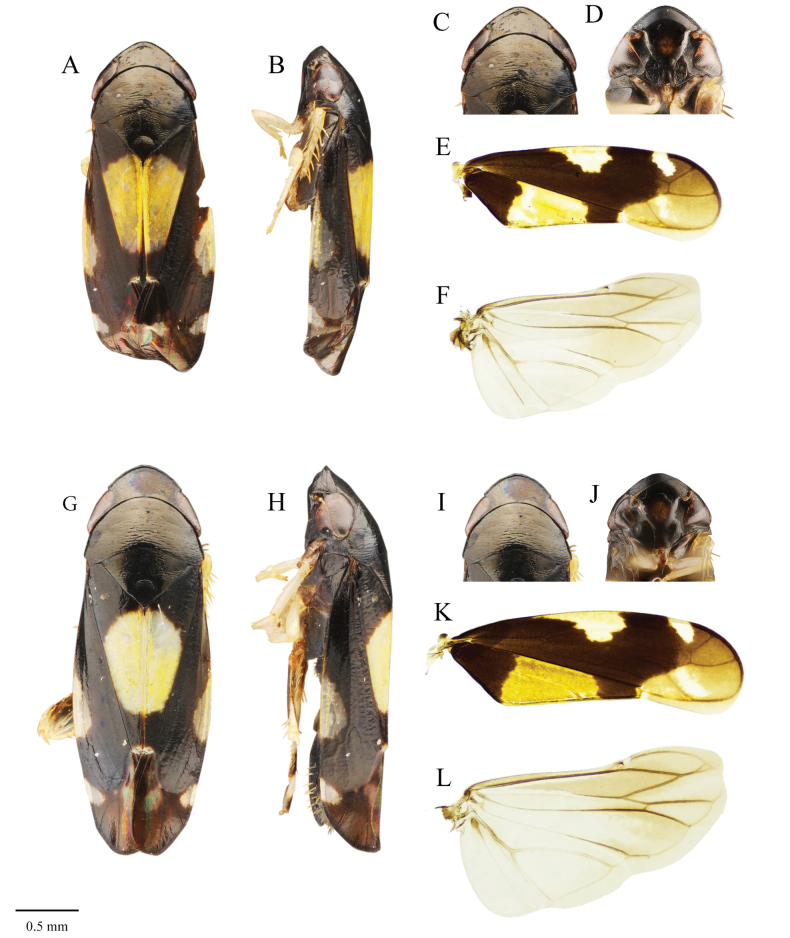
*Mukariasakaeratensis* Piraonapicha & Chomphuphuang, sp. nov., male. **A–F** (male) **G–L** (female): **A, C, G, I, J** dorsal view **B, H** lateral view **C, I** head in dorsal view, **D, J** face **E, K** forewing in dorsal view **F, L** hindwing in dorsal view. Scale bar: 0.5 mm.

**Figure 4.**
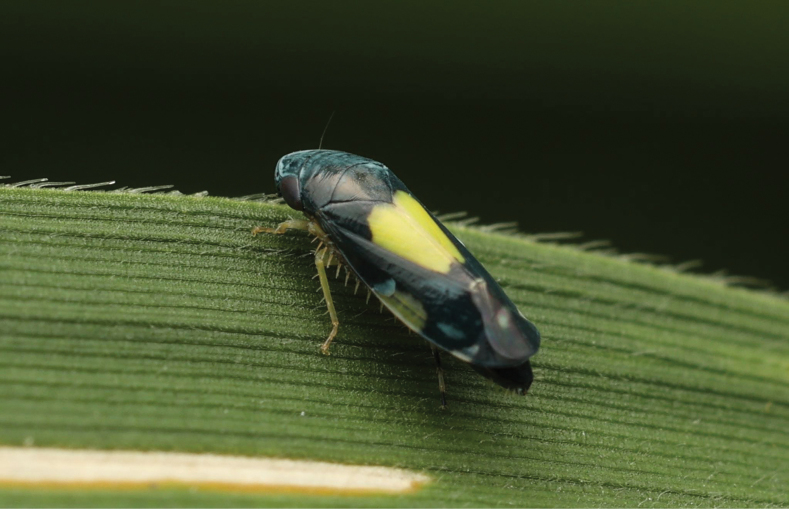
*Mukariasakaeratensis* Piraonapicha & Chomphuphuang, sp. nov., living female, dorsolateral view.

##### Remarks.

The genus *Mukaria* is distributed in the southeastern Palaearctic, Oriental and Oceanic regions. Three species were reported in Southeast Asia, i.e., *M.confersa*, *M.maculata* and *M.variabilis*. Prior to this study, there was a long gap of 63 years during which no new species of the genus were reported from Thailand. Ishihara (1961) reported a female of *M.confersa* from the northern region (Chiang Mai Province) of the country. The female of *M.confersa* differs from new species by 1) forewing apex black or varying degrees of brown, rest of wing and portion of disc of face light colored; and 2) light color spot on upper part (R-vein) of fore wing was connected with spot on lower part (M-vein) (see fig. 88 in Ishihara, 1961). The wing color and shape of the style of the new species is similar to *M.maculata* (see plate I, fig. 3 in Qiang et al. 2017), *M.splendida* (see fig. 3a in Viraktamath and Webb 2019; fig. 2g in Ramaiah et al. 2023). The new species can be distinguished from the closely related *M.maculata* by the following characteristics: 1) hook-shaped (curved-inward) of apex male aedeagus (Y-shaped of apex male aedeagus in *M.maculata*); 2) having a pair of spines pointed anteriorly at middle portion of male aedeagus on inner margin of arms (absent in *M.maculata*); and 3) length of middle area of male aedeagus as long as arms (length of middle area of male aedeagus shorter than arms in *M.maculata*). Based on the results from the phylogenetic and species delimitation analysis of the COI gene (Fig. 8), *Mukariamaculata* specimens (MG736688, MG736689, MG736690) and *Mukariamaculata* (MG736687) are placed into different clades in the phylogenetic analysis. The divergence seen in the phylogenetic tree might be due to taxonomic misidentification or inaccuracies in determining that both groups belong to *M.maculata*. These clades should be re-examined using detailed morphological, ecological, and genetic data to confirm their taxonomic assignment to *M.maculata*.

**Figure 5.**
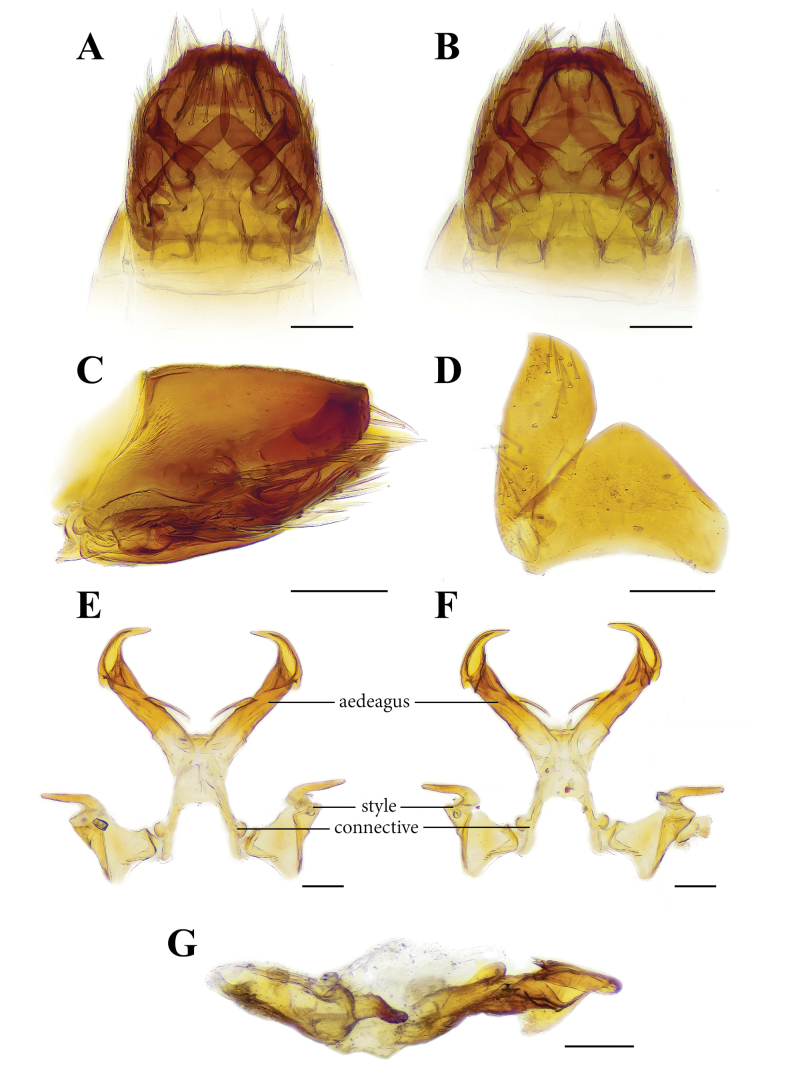
*Mukariasakaeratensis* Piraonapicha & Chomphuphuang, sp. nov., male, **A–C** genital capsule, ventral view (**A**), dorsal view (**B**), lateral view (**C**), **D** valve and subgenital plate in ventral view **E–G** style, connective and aedeagus, ventral view (**E**) dorsal view (**F**) lateral view (**G**). Scale bars: 0.2 mm (**A–C**), 0.1 mm (**E–G**).

**Figure 6.**
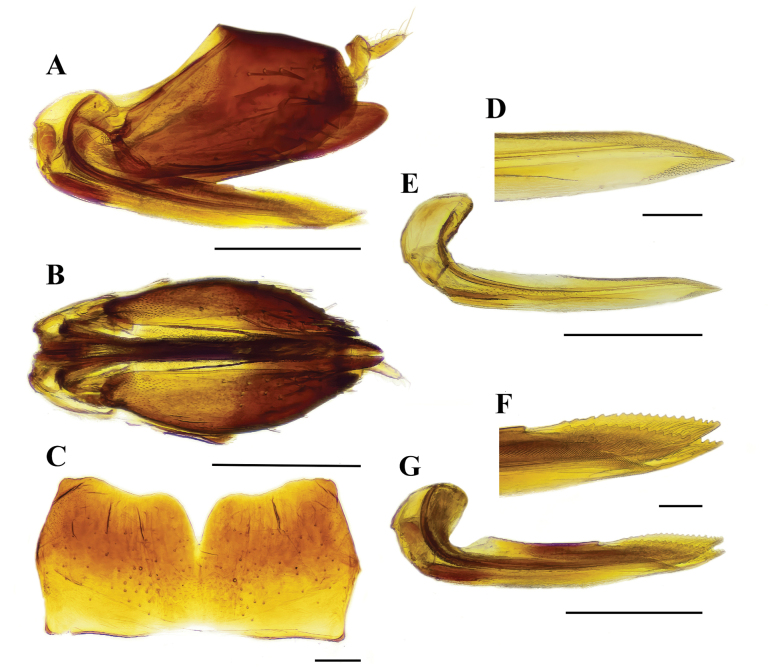
*Mukariasakaeratensis* Piraonapicha & Chomphuphuang, sp. nov., female genitalia, **A, B** genital capsule (**A**), lateral view (**B**), ventral view, **C** sternite VII **D** valvula I apex magnified in lateral view **E** valvula I in lateral view **F** valvula II, apex magnified in lateral view **G** valvula II in lateral view.

**Figure 7.**
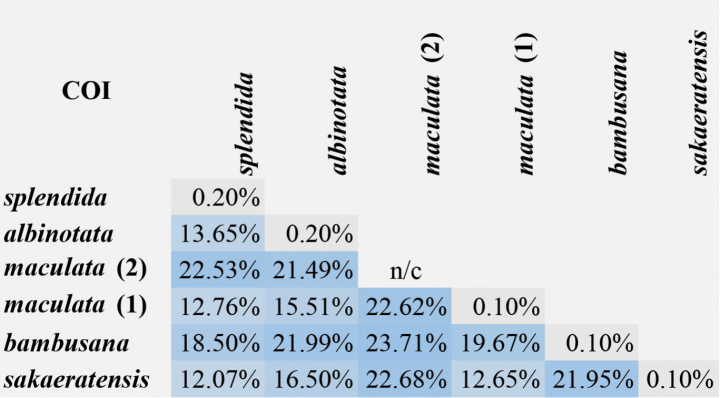
Heat map for percentage of intra- and interspecific genetic distances for *Mukaria* as determined by the Kimura 2-parameter model in COI gene.

**Figure 8.**
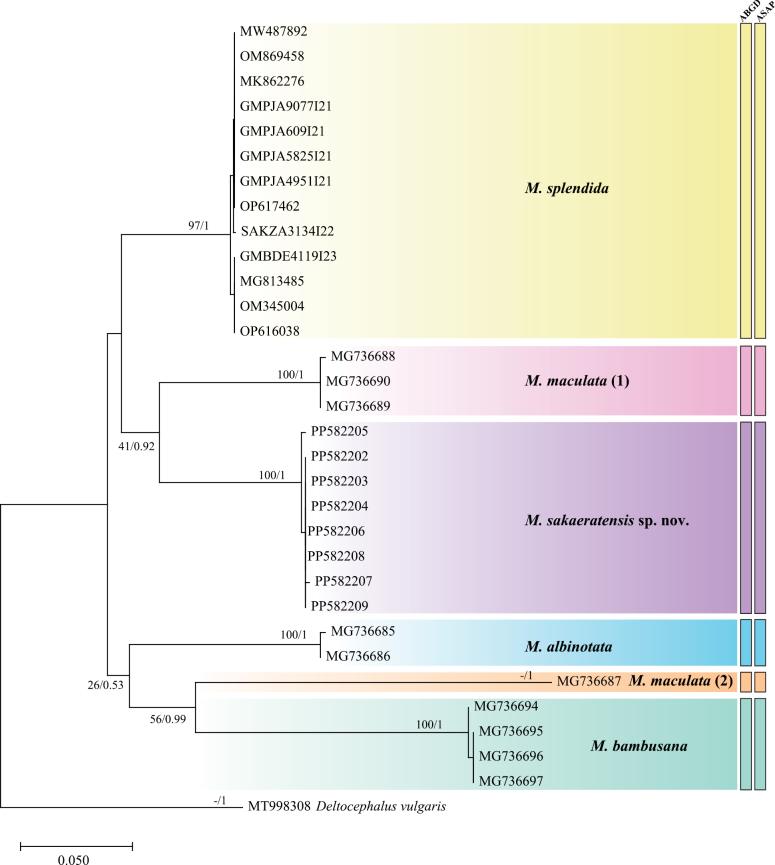
Molecular species delimitation of *Mukaria* using maximum likelihood and partial COI sequences. The tree was inferred from 592 base pairs of six species of *Mukaria* and one species of *Deltocephalus* as the outgroup. Nodal support values are bootstrap values (percentage of 1000 replicates). Node numbers represent two support values: bootstrap support from RAxML and posterior probability from Bayesian inference. The bars illustrate molecular delimitation methods, encompassing genetic distances (ABGD, ASAP). Scale bar indicates 0.050 nucleotide substitutions.

### ﻿Key to species of genus *Mukaria* from Southeast Asia based on males

**Table:** 

1	Apex of aedeagus bifurcated	**2**
–	Apex of aedeagus not bifurcated (Fig. 5E, F)	***M.sakaeratensis* sp. nov.**
2	Pair of spines near apex of aedeagus curved inward (Qiang et al. 2017: fig. 34)	***M.maculata***
–	Pair of spines near apex of aedeagus curved outward (Evans 1973: fig. 3E)	***M.variabilis***

## Supplementary Material

XML Treatment for Mukaria

XML Treatment for Mukaria sakaeratensis

## ﻿Additional information

### Author ORCIDs

Kanyakorn Piraonapicha https://orcid.org/0000-0003-3800-8625

Nithina Kaewtongkum https://orcid.org/0009-0001-8782-3055

Narin Chomphuphuang https://orcid.org/0000-0003-0738-3879

Panrak Kimsawat https://orcid.org/0009-0008-7743-8947

Kittisak Kumtanom https://orcid.org/0009-0000-9059-7872

Yudthana Samung https://orcid.org/0000-0003-4069-4257

### Data availability

All of the data that support the findings of this study are available in the main text.
